# The use of circulating cell-free tumor DNA in routine diagnostics of metastatic melanoma patients

**DOI:** 10.1038/s41598-020-61818-1

**Published:** 2020-03-18

**Authors:** Jana Knuever, Jonathan Weiss, Oana-Diana Persa, Karl Kreuzer, Cornelia Mauch, Michael Hallek, Max Schlaak

**Affiliations:** 1 Department of Dermatology and Venerology, University of Cologne, Cologne, Germany; 2 Center of Integrated Oncology (CIO), Cologne, Germany; 3 Clinic I for Internal Medicine, University Hospital of Cologne, Cologne, Germany; 4 Department of Dermatology and Allergology, Hospital of the Ludwig-Maximilians-University (LMU) Munich, Munich, Germany

**Keywords:** Melanoma, Melanoma

## Abstract

Modern advances in technology such as next-generation sequencing and digital PCR make detection of minor circulating cell-free tumor DNA amounts in blood from cancer patients possible. Samples can be obtained minimal-invasively, tested for treatment-determining genetic alterations and are considered to reflect the genetic constitution of the whole tumor mass. Furthermore, tumor development can be determined by a time course of the quantified circulating cell-free tumor DNA. However, systematic studies which prove the clinical relevance of monitoring patients using liquid biopsies are still lacking. In this study, we collected 115 samples from 47 late stage melanoma patients over 1.5 years alongside therapy-associated clinical routine monitoring. Mutation status was confirmed by molecular analysis of primary tumor material. We can show that detectable levels of circulating cell-free tumor DNA correlate with clinical development over time. Increasing levels of circulating cell-free tumor DNA during melanoma treatment with either targeted therapy (BRAF/MEK inhibitors) or immunotherapy, during recovery time or the intervals between last treatment cycle and second-line treatment point towards clinical progression before the progression becomes obvious in imaging. Therefore, this is a further possibility to closely screen our patients for tumor progression during therapy, in therapy-free phases and in earlier stages before therapy initiation.

## Introduction

Circulating cell-free tumor DNA (ctDNA) is increasingly being investigated and used as a marker in various types of tumors^1^. The basic concept is similar for all cancer types. Tumor cells release DNA molecules into the surrounding tissue, either by apoptosis or active secretion^2^. The ctDNA is then transported into the bloodstream. Circulating cell-free tumor DNA can be identified by its somatic alterations specific for the tumor type^3^. Somatic mutations at position V600 of the BRAF gene occur in 50–60% of all cutaneous melanoma and are the most common genetic alteration in this disease, although less frequent in acral and mucosal melanoma^4^. Presence of a BRAFV600 mutation renders the tumor susceptible to inhibition of the BRAF protein. In combination with a MEK inhibitor, 75% of patients respond to this treatment which leads to improved progression free and overall survival rates^5,6^. However, durable responses are rare and especially progression in the central nervous system is problematic as these metastases can evade treatment and imply a bad prognosis^7^. Alternative treatment options include immune-checkpoint inhibitors targeting either the CTLA-4 or PD-1/PD-L1 receptor. Both can now be combined as well. Clinically, it has been shown that immunotherapy is able to induce durable responses^8^. Unfortunately, there is no marker available predicting a therapeutic response before the actual start of treatment. Current clinically available methods to detect tumor progression or relapse are insensitive and often difficult to interpret, especially in patients being treated with immunotherapy. Circulating cell-free tumor DNA as a marker for tumor development has been reported as being able to fill this role in melanoma and other tumor entities^1,9^. However, most studies have focused on highly selected patient cohorts with specific treatment modalities^9^. First reports on specific high-throughput systems for detection of BRAF and NRAS ctDNA are available^10^. Lately, a proof of concept has been shown for monitoring melanoma recurrence with ctDNA for early stage melanoma (stage 0-III)^11^. Here, we aim to elucidate the implementation and impact of ctDNA in clinical routine practice for the treatment of late stage melanoma patients.

## Methods

### Patients

All patients were routinely treated at the University Hospital of Cologne for late stage IIIC-IV BRAFV600E positive cutaneous melanoma according to the American Joint Committee on Cancer (AJCC) 7^th^ edition. We collected 115 plasma samples from 47 melanoma patients from June 2015 until October 2016 (Table 1) during scheduled visits as part of the routine treatment or during the follow-up period, but only when a blood draw was otherwise necessary (Table 2). Testing for BRAFV600E in paraffin sections of primary tumors was performed in our in-house pathology department, as part of the routine treatment procedure. All patients were routinely treated in our clinic with either a BRAF inhibitor (alone or in combination with a MEK inhibitor (from October 2015)) or with immunotherapy (Pembrolizumab, Ipilimumab or Nivolumab). The combination of a PD-1 and CTLA-4 antibody was not available when samples were collected. The most suitable treatment option for each patient was discussed by an expert panel and chosen based on tumor burden, ECOG (Eastern Co-operative Oncology Group) performance status and life situation of the patient as well as previous therapies. The local clinical ethics commission (Ethics Commission of Cologne University’s Faculty of Medicine) approved the work under the number 12–163. The entire study was conducted in accordance with relevant guidelines and regulations. Informed consent was obtained from all patients and no exclusion criteria have been applied. Determination of S100 protein concentration as well as CT imaging were performed as part of the routine treatment of all patients in the cohort. CT images were analyzed in detail by a trained radiologist according to RECIST 1.1 criteria^12^. To simplify the evaluation and data correlation we pooled the therapeutic responses classified as complete remission (CR), stable disease (SD), partial response (PR) and progressive disease (PD) into two groups, namely disease control (CR, SD, PR) and disease progression (PD) (Table 2).

**Table 1:** Clinical characteristics of melanoma patients (n = 47).

Clinical characteristics of melanoma patients (n = 47)	
**Average age (years)**	55
**Sex**	
Men	26 (55.3%)
Women	21 (44.7%)
**LDH concentration (first sample)**	
>upper limit of normal	15 (31.9%)
<upper limit of normal	32 (68.1%)
**S100 concentration (first sample)**	
>upper limit of normal	22 (46.8%)
<upper limit of normal	25 (53.2%)
**AJCC tumor stage**	
IIIC	3 (6.4%)
IV M1a	5 (10.6%)
IV M1b	11 (23.4%)
IV M1c	28 (59.6%)
**Initial Treatment**	
BRAFi	13 (27.7%)
BRAFi+MEKi	17 (36.2%)
Ipilimumab	3 (6.4%)
Nivolumab	6 (12.8%)
Pembrolizumab	8 (17,0%)

*All patients were BRAFV600E positive.

**Table 2:** Correlation of imaging results with disease markers of patient samples (n = 115).

Patient No.	Sample No.	S100	ctDNA	Staging
1	1	1	0	DC
	2	1	1	
	3	1	0	DC
	4	0	0	
	5	0	0	
	6	1	0	DC
	7	0	0	
2	8	0	0	DC
	9	0	1	
	10	1	1	
	11	1	0	DC
	12	1	1	
	13	1	0	DC
3	14	0	0	DC
	15	1	1	
	16	1	1	
	17	1	0	DC
	18	1	1	
4	19	0	0	DC
	20	1	1	DC
	21	0	1	
	22	0	0	DC
	23	0	1	
5	24	1	1	DC
	25	1	0	
	26	0	0	
	27	0	0	*DP*
	28	0	1	*DP*
6	29	0	0	DC
	30	1	0	DC
	31	0	0	DC
	32	0	0	DC
7	33	0	0	DC
	34	1	0	DC
	35	0	0	
	36	0	0	
8	37	0	0	DC
	38	0	0	
	39	0	0	DC
	40	1	0	DC
9	41	0	0	DC
	42	0	1	
	43	1	1	
	44	1	0	DC
10	45	1	1	*DP*
	46	0	0	
	47	0	0	
	48	0	0	*DP*
11	49	1	1	*DP*
	50	1	0	
	51	0	0	
	52	1	0	DC
12	53	0	0	*DP*
	54	1	0	
	55	0	0	
	56	0	0	*DP*
13	57	0	0	DC
	58	1	0	
	59	1	0	DC
14	60	0	0	DC
	61	0	1	
	62	1	0	DC
15	63	1	1	*DP*
	64	0	1	
	65	1	0	DC
16	66	0	0	DC
	67	0	1	
	68	0	1	*DP*
17	69	0	0	*DP*
	70	0	0	
	71	0	0	*DP*
18	72	0	0	*DP*
	73	0	0	DC
19	74	1	0	DC
	75	0	1	
20	76	1	0	*DP*
	77	0	0	DC
21	78	1	1	DC
	79	1	0	DC
22	80	1	0	DC
	81	1	0	DC
23	82	0	0	DC
	83	0	0	
24	84	1	0	*DP*
	85	1	0	*DP*
25	86	0	0	DC
	87	0	0	
26	88	0	0	DC
	89	1	0	DC
27	90	0	0	DC
	91	0	0	
28	92	0	0	DC
	93	1	0	DC
29	94	1	0	DC
	95	0	0	
30	96	1	0	DC
	97	0	0	*DP*
31	98	0	0	DC
	99	1	0	*DP*
32	100	0	0	*DP*
33	101	1	0	DC
34	102	0	0	DC
35	103	1	1	DC
36	104	0	0	DC
37	105	1	0	DC
38	106	0	0	DC
39	107	0	0	DC
40	108	1	0	DC
41	109	0	0	DC
42	110	0	0	DC
43	111	1	0	*DP*
44	112	1	0	*DP*
45	113	1	0	*DP*
46	114	1	1	*DP*
47	115	1	1	*DP*

S100 < 0.1 ng/ml = 0

S100 > 0.1 ng/ml = 1

ctDNA not detectable = 0

ctDNA detectable = 1

DC = disease control

DP = disease progression

blank = no imaging performed

### Plasma extraction

Blood was collected in 10 ml EDTA Tubes (Sarstedt) and transported to the laboratory for plasma extraction within 3 hours. In this study, three samples reached our laboratory later than four hours and were subsequently discarded. Next, blood was centrifuged for 10 and 15 minutes, respectively, to remove the blood cells and the plasma supernatant was collected. All samples were aliquoted into 5 ml tubes and stored at −80 °C until further use.

### DNA extraction

For all DNA extractions, a free nucleic acid extraction kit was used (Qiagen, Cat No.: 61504) according to the manufacturer’s instructions. For each sample, 2 ml plasma was extracted and DNA was eluted in 25 µl of AVE elution buffer.

### Digital droplet PCR

PCR setup was applied according to manufacturer’s instructions. Each PCR was setup in 20 µl reactions containing 10 µl 2xPCR mix (Bio-Rad, Cat No.: 1863028), 1 µl of BRAFV600E (Bio-Rad, Cat. No.: dHsaCP2000027) and BRAF wt (Bio-Rad, Cat. No.: dHsaCP2000028) as well as 8 µl of sample to maximise the amount of input DNA. The whole PCR reaction was compartmentalised in ~20000 single reactions using the QX200 droplet generator (Bio-Rad, Cat. No.: 1864002). 40 µl of droplet-oil-suspension were transferred into an Eppendorf twin-tec plate and cycled on a CT1000 PCR machine (Bio-Rad, Cat. No.:1841100) according to the manufacturer’s instructions. The cycled reaction was then read on a QX200 droplet reader (Bio-Rad, Cat. No.: 1864001) and analyzed using QuantaSoft software (Bio-Rad). Each run included non-template mixes as negative controls as well as the heterozygous mutated BRAF V600E HT29 cell line DNA (genomic DNA obtained from the in-house pathology department) as positive control. Allele frequencies were calculated as counted ctDNA molecules/ctDNA + wtDNA molecules. DNA molecules per ml of plasma were calculated considering the amount of starting material, elution volume as well as volume added to the reaction.

### Statistical analysis

Statistical analysis and graph formation was performed with GraphPad Prism, version 8.2.1 (GraphPad Software, La Jolla, CA, USA). Spearman’s ρ test was applied for correlation analysis and Fisher’s exact test for the prediction value. The significance level was determined at P < 0.05.

## Results

### Detection of circulating cell-free tumor DNA and correlation to S100

**Figure 1:**
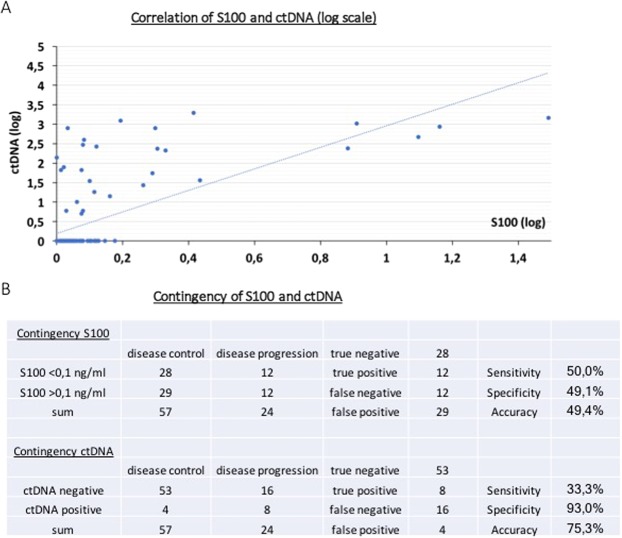
(**A**) Correlation of S100 and ctDNA. For improved visualisation depicted on a logarithmic scale. On x-axis S100 values (S100 → log_10_ (S100 + 1)), on y-axis ctDNA values (ctDNA → log_10_ (ctDNA+1)). Spearman’s ρ test: p-value < 0,0001. (**B**) Contingency of S100 and ctDNA. Sensitivity = true positive/(true positive + false negative) Specificity = true negative/(true negative + false positive) Accuracy = (true positive + true negative)/(true positive + true negative + false positive + false negative). Prediction value calculations with Fisher’s exact test: S100 not significant, ctDNA p-value 0,0047.

The cohort consists of a balanced number of male and female patients (26 vs. 21) with a mean age of 55 years at time point of blood draw (range, 23–84, Table 1). We detected measurable levels of ctDNA (BRAFV600E) in peripheral blood in 33% of the BRAF V600E positive patients included in this study (13/47) and in 25% (29/115) of all tested plasma samples. The second percentage is lower than the first, because some of the previously positively tested patients who had more than one blood draw, presented with undetectable ctDNA in the time course due to therapeutic response and thus decrease of tumor burden (Table 2). Importantly, the measured ctDNA (BRAFV600E) levels significantly correlated with the S100 levels as depicted in Fig. 1A. Correlation analysis was performed with the Spearman’s ρ test, p-value < 0,0001.

### Detectable ctDNA and S100 and correlation to imaging data

We then compared the obtained ctDNA and S100 levels with results from CT/MRI scans that were performed as part of the routine diagnostics of patients. For 80 samples, imaging data from a four-week period around the time of plasma collection was available. The imaging data was analyzed according to RECIST criteria, dichotomised as described in the methods section and correlated with the dichotomised ctDNA and S100 results (Table 2). Surprisingly, contingency analysis of S100 to predict disease control or progression showed sensitivity, specificity and accuracy of approximately 50% (Fig. 1B), Fisher’s exact test did not prove statistical significance of the S100 prediction value. When we analyzed ctDNA contingency though, it showed high specificity (93%), lower sensitivity (33.3%) and an accuracy of 75.3% (Fig. 1B). Importantly, the prediction value of ctDNA for prognosis of disease control or progression is statistically significant (p-value 0.0047, Fisher’s exact test, GraphPad Prism).

### Amount of circulating cell-free tumor DNA reflects clinical behavior

**Figure 2:**
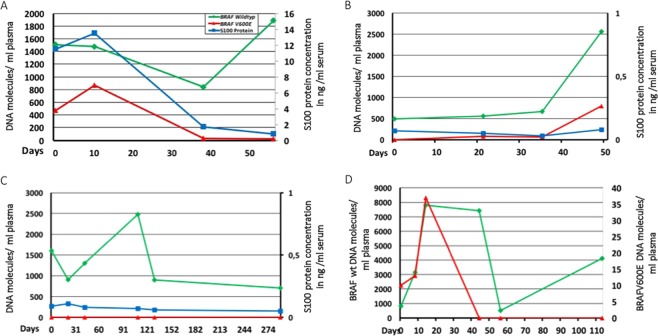
(**A**–**C**) Left y-axis = DNA molecules/ml plasma, right y-axis = S100 protein concentration in µg/ml serum; x-axis = time (days); green = BRAF wildtype ctDNA, red = BRAF V600E ctDNA, blue = S100 Protein D. Left y-axis = BRAF wt DNA molecules/ml plasma, right y-axis = BRAF V600E DNA molecules/ml plasma; x-axis = time (days); green = BRAF wildtype ctDNA, red = BRAF V600E ctDNA. (**A**) Decrease of BRAF V600E ctDNA and of S100 Protein correlate with positive treatment response. (**B**) Increase of BRAF V600E ctDNA correlates with treatment failure, while S100 Protein was never elevated. (**C**) No detection of BRAF V600E ctDNA and normal S100 Protein levels show the course of effective immunotherapy. (**D**) Debulking peak and following decrease of BRAF V600E ctDNA depict successful initiation of treatment with BRAF/MEK inhibitor.

We utilised the ability of droplet digital PCR to generate absolute counts of DNA in each sample^13^. The BRAFV600E mutation served as a marker for ctDNA and we monitored changes in the amount of ctDNA over time, alongside treatment monitoring of the patients with the commonly used serum marker S100 and parallel to routine imaging by CT/MRI, which were evaluated according to RECIST 1.1 criteria by experienced radiologists. Here, we use three exemplary patients to visualise our most common observations during the study. Figure 2A–C show three of the principal tumor development patterns. Figure 2A shows the tumor response after the initiation of treatment, by showing a debulking peak at the beginning followed by a rapid decrease to near undetectable levels. Of note, the amount of wildtype DNA (green line) spikes in the last measurement, probably due to an unrelated event such as an infection. The blue line shows the concentration of S100, a widely-used serum marker recommended by German guidelines for tumor progression in melanoma^14^. Interestingly, although the S100 concentration mimics the ctDNA measurements, the response is slower and the concentration needs more time to reach physiological concentrations. Figure 2B depicts another patient at the time point of treatment failure. There was no ctDNA detectable at first measurement; however, it quickly increased over the course of 6 weeks, showing treatment failure. Most importantly, throughout the treatment the concentration of S100 protein did not increase and was no suitable marker for this patient. Tumor relapse was further confirmed by imaging results at day 60.

Some patients respond very well to immunotherapy for extended periods of time (Fig. 2C). This patient has previously been treated with a BRAF inhibitor until relapse and then switched to immune therapy prior to our first sample collection. Over the course of the study, no ctDNA could be detected in any blood sample over the course of a full year.

### Circulating cell-free tumor DNA and S100 for disease monitoring

The S100 serum protein has been the “gold standard” for monitoring of melanoma tumor progression in the blood of patients for many years^15^. As all patients in this study were included in routine treatment, S100 protein concentrations were available for all time points where blood was taken for the detection of ctDNA. Physiological levels of S100 protein in healthy individuals are considered below 0.1 ng/ml serum^15^. Figure 2B shows one of our exemplary patients who showed no increase in S100 concentration despite massive tumor proliferation both shown by increasing amount of ctDNA as well as imaging at the time of progress. Together with the analysis of correlation between S100 and ctDNA (Fig. 1A) as well as the significant prediction value of ctDNA towards imaging of disease control/progression (Fig. 1B), this shows that ctDNA in clinical routine practice is at least as effective as S100 in response to changes of tumor load and moreover can accurately be used in patients where S100 is an insufficient marker.

## Discussion

This study aims to evaluate the practicality of circulating cell-free tumor DNA (ctDNA) as a biomarker in the routine treatment of late-stage melanoma patients and to monitor the clinical course of the disease. We evaluated 47 patients with stage IIIC-IV melanoma by comparing ctDNA levels with clinical parameters, namely CT/MRI imaging results and serum levels of S100 protein. Importantly, plasma should be extracted soon after blood collection to avoid contamination with wild type DNA deriving from i.e. leukocytes^16^. This can be problematic as it needs a reliable logistic chain. We detected ctDNA in 33% of all samples, which is lower compared to other studies. For example, the overall detection rate in 4 clinical studies looking at 746 stage IV melanoma patients was 77% when using BEAMing technology^17^. The difference is most likely due to the approach with which our samples have been collected. We collected samples at all time points throughout the treatment even when patients were in full remission and without recognizable tumor burden. When we asked the highly clinical relevant question how liquid biopsies compare to imaging results in these patients, we found a statistically significant prediction value between the two methods, which is in line with others comparing these two technologies^18^. The correlation can even further be strengthened when considering the few cases where there was a delay between the imaging scan and the blood collection. Furthermore, the RECIST criteria we used for imaging analysis in the study may not be the optimal analysis strategy. A volumetric determination of tumor burden might be a more comparable approach. We further compared our results to the commonly used S100 serum marker and observed a significant correlation between S100 and ctDNA. Most importantly, in one case we could show that the concentration of S100 was declining, even though the tumor burden rapidly increased. This is a strong indication that in some intervals liquid biopsies should be used to ensure the serum marker concentrations are correct.

In one case where time mattered due to rapid clinical progression of the patient and tissue analysis via pathology was technically difficult and long-lasting, we could use the results of liquid biopsy (high amount of BRAFV600E mutated DNA in the plasma compartment) to justify the treatment start with targeted therapy. She immediately responded well to the BRAF/MEK inhibitor which could be observed both clinically and by a decrease of ctDNA in our frequent follow-up liquid biopsies (Fig. 2D). This saved the patient valuable time and suffering.

In general, blood was collected only when otherwise necessary to avoid invasive diagnostics for our patients. This led to inconsistent sample numbers per patient and irregular time points of analysis. However, because we did not structure the analysis but rather collected the results retrospectively in a non-biased manner, it is thus not influenced by cohort criteria or observer’s bias. As all our patients receive regular staging with CT/MRI, usually every 3 months, we could still correlate the blood analysis with frequent imaging data. Future studies will be necessary to assess the prognostic importance of liquid biopsies regarding progression-free and overall survival of melanoma patients.

This study does not claim to be a controlled trial or a complete, self-contained investigation but rather it reflects transparent real-life data from the clinic, retrospectively evaluated to judge for significance but also practicality of liquid biopsies. Taken together, our study shows that liquid biopsies are an important diagnostic tool to monitor the tumor response towards treatment and accurately reflects the clinical course of the patient suggesting liquid biopsies could reduce the number of imaging diagnostics in the future.

## Data Availability

The datasets generated and analysed during the current study are available from the corresponding author on reasonable request.
